# Quantum transport simulations of graphene nanoribbon devices using Dirac equation calibrated with tight-binding *π*-bond model

**DOI:** 10.1186/1556-276X-7-114

**Published:** 2012-02-10

**Authors:** Sai-Kong Chin, Kai-Tak Lam, Dawei Seah, Gengchiau Liang

**Affiliations:** 1 Institute of High Performance Computing, A*STAR, 1 Fusionopolis Way, #16-16 Connexis, Singapore 138632, Singapore; 2 Department of Electrical and Computer Engineering, National University of Singapore, Singapore 117576, Singapore

**Keywords:** graphene nanoribbons, Dirac equation, quantum transport, non-equilibrium Green's function

## Abstract

We present an efficient approach to study the carrier transport in graphene nanoribbon (GNR) devices using the non-equilibrium Green's function approach (NEGF) based on the Dirac equation calibrated to the tight-binding *π*-bond model for graphene. The approach has the advantage of the computational efficiency of the Dirac equation and still captures sufficient quantitative details of the bandstructure from the tight-binding *π*-bond model for graphene. We demonstrate how the exact self-energies due to the leads can be calculated in the NEGF-Dirac model. We apply our approach to GNR systems of different widths subjecting to different potential profiles to characterize their device physics. Specifically, the validity and accuracy of our approach will be demonstrated by benchmarking the density of states and transmissions characteristics with that of the more expensive transport calculations for the tight-binding *π*-bond model.

## 1 Introduction

Recent progress of graphene nanoribbon (GNR) fabrication has demonstrated the possibility of obtaining nano-scale width GNRs, which have been considered as one of the most promising active materials for next generation electronic devices due to their unique properties such as bandgap tunability via controlling of the GNR width or subjecting GNR to external electric/magnetic fields [1-5]. Device simulations play an important role in providing theoretical predictions of device physics and characteristics, as well as in the investigation of device performance, in order to guide the development of future device designs. Due to the nano-scale structures of GNRs, however, semi-classical treatments of carrier transport [6], which are the mainstay of microelectronics, are no longer valid. As a result, quantum transport formalism based on models incorporating detailed atomic structures, such as the *ab-initio types *[7-9], is needed for the proper simulation of these materials. Unfortunately, a full-fledge *ab-initio *atomistic model for carrier transport simulation is still very computationally expensive and impractical even with the latest state-of-the-art computing resources. In this study, we therefore develop an efficient model in which a tight-binding Dirac equation (TBDE), calibrated with parameters from the tight-binding *π*-bond model (TB-*π*) [10-13], is used together with the non-equilibrium Green's function approach (NEGF) [14] to investigate transport properties of GNRs. We compare the density of states, *DOS*(*E*), and the transmission, *T(E)*, of selected GNR devices for our TBDE model with that of the more expensive TB-*π *model. Good agreement is found within the relevant energy range for flat band, Laplace and single barrier bias condition. We believe that our model and calibrated data for a side selection of GNR widths presented in this article provided researchers in the quantum transport an accurate and practical framework to study the properties, particularly quantum transport in arbitrary bias conditions, of GNR-based devices.

## 2 Model

**Figure 1:**
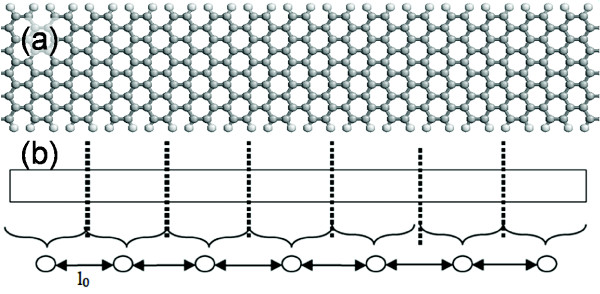
**Schematic representation of mapping of (a) a real-space two-dimensional GNR to (b) the one-dimensional Dirac Equation model with two degrees of freedom per effective cell of length ℓ_0_**.

The Hamiltonian based on the Dirac equation [15,16] for graphene is given as:

(1) $$H=\left[\begin{array}{cc} U\left(x\right) & v_{F}\left(px-ip_{y}\right)\\ v_{F}\left(px+ip_{y}\right) & U\left(x\right) \end{array}\right],$$

where *p*_*μ *_= --*iћ*∂_*μ *_is the momentum for the direction *μ *= {*x*,*y*}, *v*_*F *_is the Fermi velocity of graphene at the Dirac points (fixed at 10^6 ^ms^-1^) and *U*(*x*) is the on-site potential. Due to the 1D property of GNRs, the finite difference approach can be used along the transport direction (*x*) of GNRs and the Hamiltonian (*h*_*n*_) at each site *n*, and its backward (*h*_-_) and forward (*h*_+_) couplings with its neighbors (separated by a uniform spacing *l*_0_) for a particular subband mode *k*_*y*_, can be written as:

(2) $$\begin{array}{l} h_{n}=\left[\begin{array}{cc} U_{n} & -i\hbar v_{F}k_{y}\\ i\hbar v_{F}k_{y} & U_{n} \end{array}\right]\\ h_{-}={\left(h_{+}\right)}^{†}=\frac{i\hbar v_{F}}{2l_{0}}\left[\begin{array}{cc} 0 & 1\\ 1 & 0 \end{array}\right] \end{array}$$

where *l*_0 _is the effective 1D cell size as a result of the discretized Hamiltonian in (2). Figure 1a shows the schematics for real-space graphene and Figure 1b the 1D GNR model associated with (2). For an infinitely long GNR with uniform *U*_0_, the Bloch waves solutions are valid and the dispersion relation, *E*(*k*_*x*_,*k*_*y*_), for (2) is

(3) $$E\left(k_{x},k_{y}\right)=U_{0}\pm \left(\frac{\hbar v_{F}}{l_{0}}\right)\sqrt{{\left(k_{y}l_{0}\right)}^{2}+{\text{sin}}^{2}\left(k_{x}l_{0}\right).}$$

where for a fixed *k*_*y *_the positive and negative signs denoting the conduction and valence bands, respectively. In the absence of external potential (*U*_0 _= 0) and in the limit of large GNR width at which $\left|\vec{k}\right|$ is small, (3) gives the linear dispersion for graphene $E\left(k\right)=\pm \hbar v_{F}\left|\vec{k}\right|$. The energy bandgap of a certain width, and hence *k*_*y*_, is given by *E*_*g *_= 2*ћv*_*F*_*k*_*y *_at *k*_*x *_= 0.

For non-equilibrium situations, we have to calculate the device retarded Green's function *G*(*E*) for a particular energy *E *for the Hamiltonian in (2). Assuming the potential energies at the equilibrium source and drain are *U*_*s *_and *U*_*d*_, respectively, and there are *N *lattice points in the device region, the *G*(*E*), of matrix size 2*N *× 2*N*, is given by *G*(*E*) = [*EI*_2*N *_- *H *- *Σ*_*s *_- *Σ*_d_]^-1^, where the 'self-energies' *Σ*_*s *_and *Σ*_*d *_are associated with the effects of the semi-infinitely long source and drain [14]. Consider the self-energy of the drain (specified by the Hamiltonian *H*_*d *_of size 2*M *× 2*M*, where *M *is an arbitrary number of lattice points with spacing *l*_0 _spanning the drain), defined in the NEGF framework [14] by $\Sigma_{d}\equiv \tau_{+}G\left(E\right)\tau_{-}$, where the drain Green's function, $G\left(E\right)\equiv {\left(E-H_{d}\right)}^{-1}$, is also of the size 2*M *× 2*M*, and *τ*_- _= (*τ*_+_)^† ^is the coupling matrix (of size 2*M *× 2*N*) between the device and drain, which ends and starts at lattice points *n *= - 1 and 0, respectively. However, the only non-zero component of *τ*_± _is that of *h*_± _across the *n *= - 1 and 0 interface, and hence only the 2 × 2 drain surface Green's function $G_{0,0}$, makes non-trivial contribution to *Σ*_*d*_, i.e., $\sigma_{d}=h_{+}G_{0,0}h_{-}$ is the only non-zero 2 × 2 submatrix, associated with lattice point *n *= - 1, of *Σ*_*d *_(of size 2*N *× 2*N*). Using the identity $\left(EI_{2M}-H_{d}\right)G=I_{2M}$ for the drain region (*n *≥ 0), the system of equations for the dimensionless Green's function *G *can be written as

(4) $$\omega^{\left(0\right)}G_{0,0}-h_{+}G_{1,0}=I_{2},\;n=0$$

(5) $$-h-G_{n-1,0}+\omega^{\left(0\right)}G_{n,0}-h_{+}G_{n+1,0}=0,\;n\geq 1$$

where *ω*^(0) ^= *EI*_2 _- *h*_0 _is independent of sites inside the drain with uniform *U*_*d*_. One can iteratively substitute $G_{n>0,0}$ (second term) in (5) with the same in (4) so that after ℓ ≥ 1 number of iterations, (4) and (5) can be rewritten as [17]

(6) $$\omega_{0}^{\left(0\right)}G_{0,0}=I_{2}+\alpha^{\left(\ell \right)}G_{2^{\ell },0}$$

(7) $$\omega^{\left(\ell \right)}G_{2^{\ell }m,0}=\alpha^{\left(\ell \right)}\left[G_{2^{\ell }\left(m-1\right),0}+G_{2^{\ell }\left(m+1\right),0}\right],m\geq 1$$

where

(8) $$\alpha^{\left(0\right)}=h_{-}$$

(9) $$\beta^{\left(0\right)}=h_{+}$$

(10) $$\alpha^{\left(\ell \right)}=\beta^{\left(\ell \right)},\;\ell \geq 1$$

(11) $$\alpha^{\left(\ell \right)}=\alpha^{\left(\ell -1\right)}\left[\omega^{\left(\ell -1\right)-1}\right]\alpha^{\left(\ell -1\right)}=\Lambda^{\left(\ell \right)}\left(\lambda \right)\omega^{\left(0\right)}$$

(12) $$\omega^{\left(\ell \right)}=\omega^{\left(\ell -1\right)}-2\alpha^{\left(\ell \right)}=\Omega^{\left(\ell \right)}\left(\lambda \right)\omega^{\left(0\right)}$$

(13) $$\omega_{0}^{\left(\ell \right)}=\omega_{0}^{\left(\ell -1\right)}-\alpha^{\left(\ell \right)}=\Omega_{0}^{\left(\ell \right)}\left(\lambda \right)\omega^{\left(0\right)}.$$

The prefactor $\lambda =e^{ik_{x}l_{0}}$ is such that *k*_*x *_is related to *E *via (3). The integer *m *≥ 0 labels the surviving lattice points with spacing 2 ^ℓ^*l*_0_. The effects of the eliminated nodes after *ℓ *number of iterations are taken into account in terms of "renormalized" couplings *α*^(*ℓ*) ^and *β*^(*ℓ*)^, (which happens to be equal in this model) and site energies (*ω*^(*ℓ*) ^at site index 2^ℓ^*m *with *m *≥ 1 and $\omega_{0}^{\left(\ell \right)}$ at *m *= 0, respectively). The symmetries of *h*_0 _and *h*_± _in (2) resulted in *α*^(*ℓ*)^, *ω*^(*ℓ*) ^and $\omega_{0}^{\left(\ell \right)}$ each directly proportional to the "bare" energy *ω*^(0) ^for all *ℓ *≥ 1, with their respective coefficients *Λ*^(*ℓ*)^, *Ω*^(*ℓ*)^, and $\Omega_{0}^{\left(\ell \right)}$ as scalar functions dependent on λ only. We show by induction that for all *ℓ *≥ 1,

(14) $$\Lambda^{\left(\ell \right)}\left(\lambda \right)=\frac{1}{1-\lambda^{2}}\left[\frac{\lambda^{2^{\ell }}}{\sum_{j=0}^{2^{\ell }-1}\left(-1\right)j\lambda^{2j}}\right],$$

(15) $$\Omega^{\left(\ell \right)}\left(\lambda \right)=\frac{\lambda }{1-\lambda^{2}}\left[\frac{1+\lambda^{2^{\ell +1}}}{\sum_{j=0}^{2^{\ell }-1}\left(-1\right)j\lambda^{2j}}\right],$$

(16) $$\Omega_{\ell }^{0}\left(\lambda \right)=\frac{\lambda }{1-\lambda^{2}}\left[1+\frac{\lambda^{2^{\ell +1}}}{\sum_{j=0}^{2^{\ell }-1}\left(-1\right)j\lambda^{2j}}\right]$$

uniquely satisfy (11), (12), and (13). Since we are interested in the retarded Green's function (i.e., *E *→ *E *+ *iη*) for an infinitesimally small energy *η *> 0, the condition imposed on the propagating waves is such that |*λ*| ≈ 1 - (*l*_0_/*ћv*_*g*_)*η *< 1, where *v*_*g *_≡ *ћ*^-1 ^(∂*E*/*∂k*_*x*_) > 0 is the relevant group velocity [18,19]. Expanding in terms of *λ *and taking the limit *ℓ *→ ∞, (14), (15), and (16) give *A*^(∞) ^= 0, *Ω*^(∞) ^= (1 + *λ*^2^)/(1 - *λ*^2^), and $\Omega_{0}^{\left(\infty \right)}=1/\left(1-\lambda^{2}\right)$, respectively. The exact value of $G_{0,0}$, in the limit of *ℓ *→ ∞ in (6), is now given by

(17) $$G_{0,0}=\frac{4\lambda^{2}}{\lambda^{2}-1}{\left(\frac{l_{0}}{\hbar v_{F}}\right)}^{2}\left[\begin{array}{cc} E-U_{d} & -i\hbar v_{F}k_{y}\\ i\hbar v_{F}k_{y} & E-U_{d} \end{array}\right]$$

Similar argument can be applied at the source-channel interface where the analog source-side counterpart of $G_{0,0}$ takes the same form as (17) with *U*_*s *_replacing *U*_*d*_. Therefore, the only non-zero 2 × 2 submatrices for *Σ*_[*s, d*] _are

(18) $$\sigma_{\left[s,d\right]}=\frac{\lambda^{2}}{\lambda^{2}-1}\left[\begin{array}{cc} E-U_{\left[s,d\right]} & i\hbar v_{F}k_{y}\\ -i\hbar v_{F}k_{y} & E-U_{\left[s,d\right]} \end{array}\right]$$

**Figure 2:**
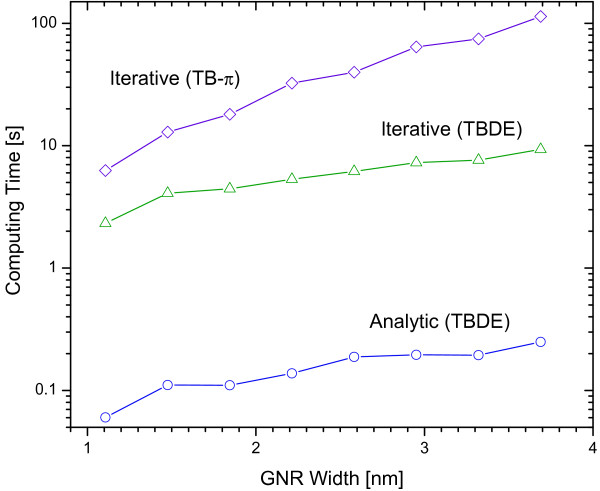
**The total computing time for calculating a series of $G_{0,0}$(*E*) for all relevant modes in - 1 ≤ *E *≤ 1 eV with 0.001 eV spacing using analytic (○) and iterative (Δ) methods in the TBDE model for different GNR width**. The iterative method takes about 40 × longer than that of analytic method. Included for comparison is the total time to calculate the corresponding surface Green's functions calculated using iterative method in the TB-*π *model (◊).

In the past, (6)-(13) are evaluated iteratively to calculate $G_{0,0}$, and hence *Σ*_[*s, d*] _[13,17]. In this study, we have shown that (6)-(13) can be solved analytically for the Dirac form in (2) and that significant computational saving and accuracy can therefore be achieved by directly using (18) instead of numerically iterating (6)-(13). Figure 2 shows that the total computing time to calculate all the relevant modes of $G_{0,0}$(*E*) for *E *∈ [-1,1] eV with spacing of 0.001 eV via analytical, i.e., (17), and iterative means, i.e., (6)-(13) for a range of GNR width on a typical duo core PC using MATLAB. The time needed to calculate $G_{0,0}$ using the iterative method is about 40× larger than that of the analytic method over the entire range of the GNR width considered. In general, it is observed that the computing time increases with the GNR width for both analytical and iterative methods because the number of modes also increases with the width. (See Table 1.) Figure 2 also shows, as a comparison, the corresponding total computing time for calculating the all relevant surface Green's functions (via iterative method) for the same set of GNR width in TB-*π *model. This time is much larger than that of the TBDE, between about 100× (at 1.1 nm width) and 455× (for 3.8 nm width) that of the analytic method of TBDE. Therefore the computational saving from using our analytic results for the surface Green's function, (17), is compelling. The computing saving will be even more apparent in more realistic quantum transport calculations in which the NEGF and Poisson equation are solved iteratively to achieve self-consistent solutions.

**Table 1:** Results of best-fit *l*_0 _(for their respective subbands) to be used for our TBDE model for GNRs of different widths

Family 3p	*E*_*g*_ (eV)	Subbands (eV) [*l*_0 _(nm)]	Family 3*p *+1	*E*_*g*_ (eV)	Subbands (eV) [*l*_0 _(nm)]
W12	1.22	0.612 [2.300], 0.859 [1.860]	W10	0.874	0.437 [1.960], 1.273 [2.712], 1.808 [1.650]

W15	0.95	0.477 [2.258], 0.682 [1.917], 1.654 [1.675], 1.760 [3.150]	W14	0.675	0.337 [2.002], 0.966 [2.528], 1.446 [1.725]

W23	0.66	0.331 [2.230], 0.482 [1.974], 1.208 [2.741], 1.209 [1.800], 1.831 [1.710]	W18	0.549	0.275 [2.031], 0.778 [2.442], 1.201 [1.832], 1.914 [3.150], 1.963 [1.630]

With *G*(*E*) now specified, the *DOS*(*E*), *T*(*E*), line charge density (*ρ*_1*D*_) and total current (*I*_*t*_) can be obtained, respectively [20], via

(19) $$DOS\left(E\right)=-\frac{1}{2\pi }\text{Tr}\left[G\left(\Gamma_{s}+\Gamma_{d}\right)G^{†}\right],$$

(20) $$T\left(E\right)=\text{Tr}\left[\Gamma_{s}G\Gamma_{d}G^{†}\right],$$

(21) $$\rho_{1d}=\underset{sb}{\sum }\int \frac{dE}{2\pi l_{0}}\text{Diag}\left[G\left(\Gamma_{s}f_{s}+\Gamma_{d}f_{d}\right)G^{†}\right],$$

(22) $$I_{t}=\frac{2e}{h}\underset{sb}{\sum }\int dE\left[f_{s}-f_{d}\right]T\left(E\right),$$

where $\Gamma_{\left[s,d\right]}\equiv i\left(\Sigma_{\left[s,d\right]}-\Sigma_{\left[s,d\right]}^{†}\right)$, *f*_*s*,*d*_(*E*) is the Fermi function at either the source or drain, *Σ*_*sb *_denotes sum over the subbands, Diag[⋯] and Tr[⋯] denote the diagonal and the trace of a square matrix, respectively.

## 3 Results and discussions

**Figure 3:**
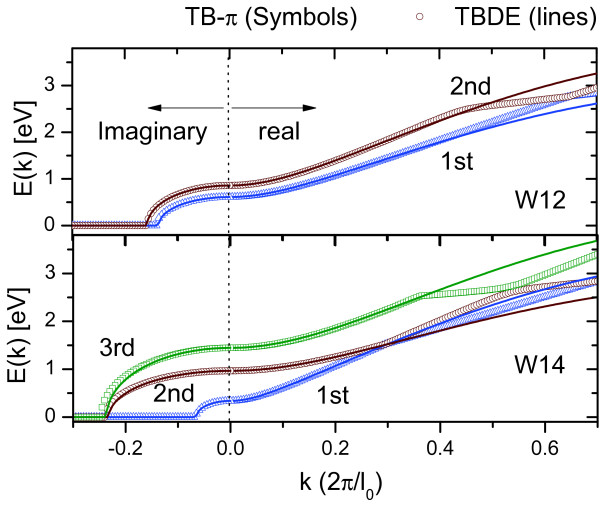
**The *E*(*k*) calculated from the TBDE matching that of the TB-*π *with different best fit *l*_0 _for different subbands for the (a) W12 and (b) W14 devices**. Only conduction bands for *E *≥ 0 are shown. The valance bands are symmetric about *E *= 0.

**Figure 4:**
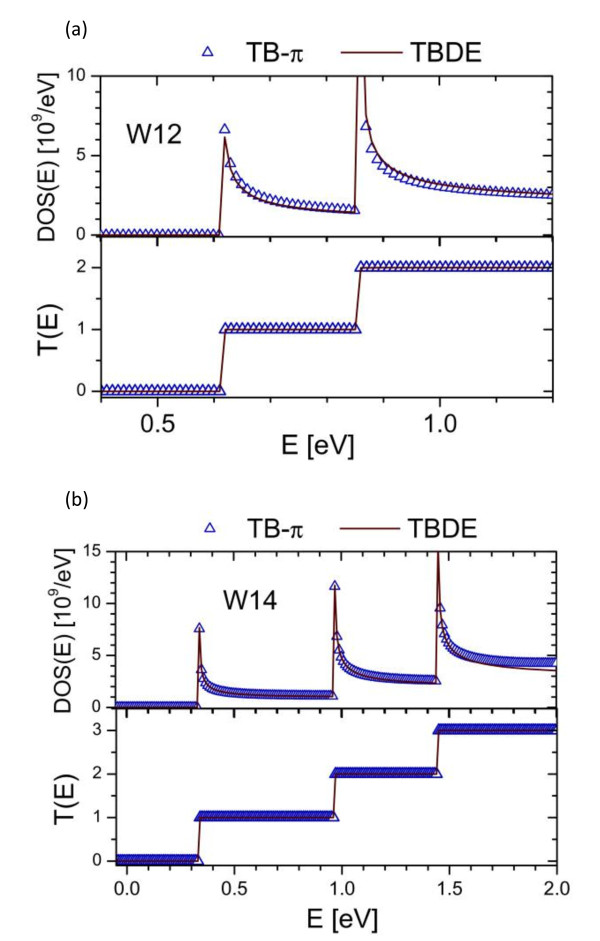
**The *DOS*(*E*) and *T*(*E*) of (a) W12 and (b) W14 devices with the best-fit *l*_0 _(from *E(k) *calculations) at *U *= 0 agreeing to that of the TB-*π *model**. Both the *DOS*(*E*) and *T*(*E*) are symmetric about *E *= 0.

To incorporate the material details of GNR into the TB-*π *model, we first fit (3) of different GNR widths with that of the TB-*π *model, which is widely used to calculate the bandstructures of GNR, for a flat potential (i.e., *U *= 0). Both real and imaginary parts of (3) are fitted for multiple subbands with different values of *l*_0 _for a particular GNR system. Figure 3 shows the comparisons of *E*(*k*) for the GNRs with width 1.0 nm and 1.4 nm, labeled as W10 and W14, respectively. At larger *k*, the *E*(*k*) calculated using (3) deviated from the that of the TB-*π *model. This is expected as the TBDE model for GNR is most accurate near the Dirac points at small *k*[15]. Since we are interested in semiconductor properties of GNRs, only the wide bandgap armchair GNRs (families with indices of *m *= 3p and 3*p*+1) [8,21] are considered here. The GNRs associated with *m *= 3*p *+ 2 have *E*_*g *_that are too small and are not considered here. Table 1 shows the best-fit *l*_0 _at different subbands for the *m *= 3*p *and *m *= 3*p *+ 1 GNRs obtained under this study. With these calibrations, the adequate bandstructure details based on TB-*π *model can be incorporated in the TBDE model. Figure 4 compare the *DOS*(*E*) and *T*(*E*) for the same W12 and W14 systems using TBDE model (with the fitted-lo values from Table 1) and that of the TB-*π *model. The very good agreements of results between the two models is a good first step to demonstrate the validity of the TBDE model in tackling quantum transport problems at which accurate *T*(*E*) and *DOS*(*E*) are the keys.

**Figure 5:**
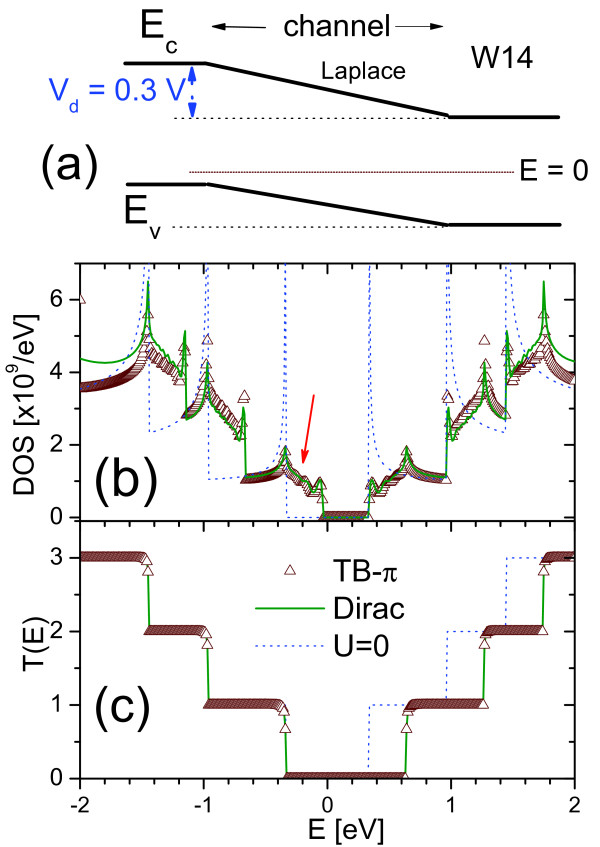
**The *DOS*(*E*) and *T*(*E*) of a simple Laplace Potential**. **(a) **Schematic of a simple Laplace potential profile with a bias of 0.3 V across the GNR channel. **(b) **The resulting *DOS(E) *versus *E *with red arrow indicating the new addition of *DOS(E) *due to the upward movement of valence band-edge by 0.3 eV. **(c) ***T(E) *versus E. Results for *U *= 0 and that calculated by TB-*π *are also included for comparison.

To apply the NEGF-TBDE to more realistic transport situations, one needs to solve the NEGF-TBDE under bias potentials. For a Laplace potential (with a bias of 0.3 V), as shown in Figure 5a, the *DOS*(*E*) and *T*(*E*) for the W14 GNR are shown in Figure 5b,c, respectively. The corresponding TB-*π *results and that of TBDE model with *U *= 0 are also included for reference. As shown in Figure 5a, the 0.3 V bias is achieved by shifting the conduction and valence bands upwards relative to those at the drain. As the highest valence band-edge (*E*_*v*_) (at source) shifted up by 0.3 eV, the onset of *DOS(E) *for *E *< 0 also creeped up into the original forbidden zone (with *U *= 0) by about 0.3 eV as indicated by arrow in Figure 5b. The positions of the *DOS*(*E*) associated with the higher subbands have also moved up the energy scale relative to those for *U *= 0. However, the onset of *DOS*(*E*) for *E *> 0 has not been affected significantly by the Laplace setup because the lowest conduction band-edge, which is at the drain, is still intact at *E *= *E*_*g*_/2. Although the forbidden zone for *DOS*(*E*) has narrowed as indicated in Figure 5b, the forbidden zone for *T*(*E*) has actually widen, as shown in Figure 5c, with the onset of non-zero *T*(*E*) for *E *> 0 receding upwards by about 0.3 eV as indicated by the arrow, but unchanged *T*(*E*) for *E *< 0. This is because from carriers are only unhindered source-to-drain only at *E *>*E*_*g*_/2 + 0.3 eV and *E *<*E*_*g*_/2. The newly addition of *DOS*(*E*) at the source-side valence has no state of comparable *E *to connect to in the channel and drain and hence does not contribute to *T*(*E*).

**Figure 6:**
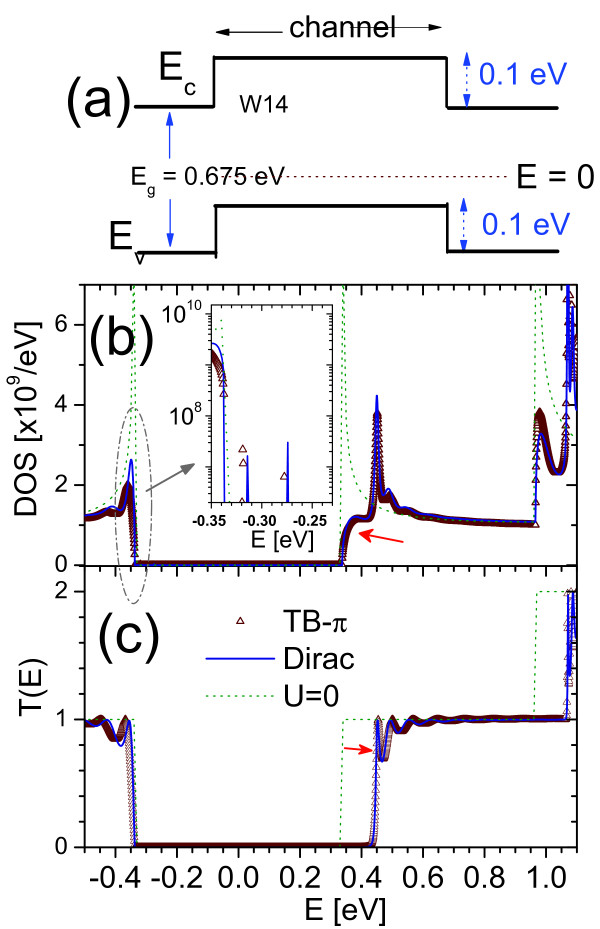
**The *DOS*(*E*) and *T*(*E*) of a rectangular barrier**. **(a) **Schematic of a rectangular barrier of height 0.1 eV across the W14 GNR channel. **(b) **The *DOS(E) *with red arrow indicating region of reduced *DOS(E) *due to the introduction of the barrier at channel. The region near *E *= *E*_*g*_/2 (as indicated) is magnified as inset with *DOS(E) *in log-scale. Two discrete bound states, created by the inverted well at valence band-edge, as shown in (a). (**c**) The *T(E) *with red arrow indicates the receding *T(E) *away from *E*_*g*_/2 due to the 0.1 eV barrier. Results for *U *= 0 and that calculated from TB-π are also included for comparison.

Next, we subjected the W14 GNR to a rectangular barrier of 0.1 eV in the channel as shown in Figure 6a. The resulting *DOS(E) *and *T(E) *are shown in Figure 6b,c, respectively, with that of TB-*π *model and *U *= 0 included for comparison. As expected, the onset of both *DOS(E) *at the conduction and valence ranges have not changed because the lowest *E*_*c *_and highest *E*_*v*_, at -*E*_*g*_/2, and *E*_*g*_/2, respectively, have not been changed by the introduction of the barrier potential compared to that of *U *= 0. However, it is observed that the magnitude of *DOS(E) *just above *E *= *E*_*g*_/2 was reduced significantly due to the lost of states in the channel region dominated by the barrier. The inverted well of depth 0.1 eV at the channel valence band-edge is expected to accommodate some discrete bound states. However, the *DOS(E) *associated with them may be too sharp to be captured, or partially captured by the *E *grids being used. This expectation is borne out by the inset of Figure 6b, which shows the log-scale of the *DOS(E) *in the vicinity of *E *= -*E*_*g*_/2. Two discrete bound states, with the heights of their *DOS(E) *partially captured, are discernible within the inverted well energy range of within 0.1 eV above -*E*_*g*_/2. As for *T*(*E*), the carriers are unhindered source-to-drain only for *E *>*E*_*g*_/2 + 0.1 eV and *E *< -*E*_*g*_/2 eV and hence those boundaries marked the onset of *T*(*E*), as shown in Figure 6c. The bound states created by the inverted well in the channel region do not contribute to *T(E) *as there are no states of comparable energies both at the source and drain to connect to them.

In both the Laplace and rectangular barrier potential profiles, the *DOS*(*E*) and *T*(*E*) for our TBDE model are in satisfactory agreement with that calculated from TB-*π *model within about 1.5 eV around the mid-gap. At higher energies, significant deviations in the *DOS*(*E*) and *T*(*E*) are consistent with the discrepancies we observed in *E*(*k*) (as shown in Figure 3b), as discussed earlier. Nonetheless, these deviations are limited to the high-energy range that is of little relevance to the electron transport in GNR devices. Therefore, our TBDE approach is expected to be valid and as a practical and efficient alternative to TB-*π *for studying carrier transport involving arbitrary self-consistent electrostatic potentials for device simulations [22,23].

## 4 Conclusion

We developed a tight-binding Dirac equation for practical and accurate numerical investigation of the electron transport in GNR devices. Based on our knowledge, this is the first time that the surface Green's function arises from applying the Dirac equation in NEGF framework is calculated exactly and hence can be used to achieve significant savings in NEGF calculations. The TBDE model is calibrated, with the appropriate parameters (*v*_*F *_= 10^6 ^ms^-1 ^and *l*_0_), to match the relevant bandstructure details as that of the TB-*π *model, especially near the Dirac points. The best-fitted *l*_0 _for a selected set of GNR widths are also presented for use. We show that the *DOS*(*E*) and *T(E) *calculated by our calibrated TBDE model can produce very good agreement with those that are calculated by the more expensive TB-π model for the flat, Laplace, and rectangular barrier potentials. These cases validate the accuracy of the TBDE model and provided good confidence that the model can be used as a practical and accurate starting point for quantum transport of GNR-based devices where non-equilibrium and arbitrary electrostatic potentials are involved.

## Competing interests

The authors declare that they have no competing interests.

## Authors' contributions

S-KC conceived the possibility of an analytic expression for the surface Green's function in Dirac equation and performed the calculations. He also prepared the manuscript. K-TL partially wrote the codes for the Dirac equation and together with DS carried out the numerical studies and analysis. GL conceived of the overall study, designed most of the codes, and participated in the analysis. All authors read and approved the final manuscript.
